# Supplementary material for: Effect of Tube Diameters and Functional Groups on Adsorption and Suspension Behaviors of Carbon Nanotubes in Presence of Humic Acid
Source: Nanomaterials (Basel). 2022 May 7;12(9):1592. doi: 10.3390/nano12091592 (PMC9100522; doi:10.3390/nano12091592)
Supplement: Supplementary file 1 [file nanomaterials-12-01592-s001.zip › nanomaterials-1684545-supplementary.pdf]

Supplementary Materials

Effect of tube diameters and functional groups on adsorption and suspension behaviors of carbon nanotubes in presence of humic acid

Mengyuan Fang [1,2,†], Tianhui Zhao [1,†], Xiaoli Zhao [1], Zhi Tang [1,*], Shasha Liu [3], Junyu Wang [1], Lin Niu [1] and Fengchang Wu [1]

1 State Key Laboratory of Environmental Criteria and Risk Assessment, Chinese Research Academy of Environmental Sciences, Beijing 100012, China; fangmengyuanfmy@163.com (M.F.); zth2512@163.com (T.Z.); zhaoxiaoli_zxl@126.com (X.Z.); jy.wong@foxmail.com (J.W.); 18233271321@163.com (L.N.); wufengchang@mail.gyig.ac.cn (F.W.)
2 College of Geoexploration Science and Technology, Jilin University, Changchun 130026, China
3 School of Energy and Environmental Engineering, University of Science and Technology Beijing, Beijing, 100083, China; liushashajida@163.com
* Correspondence: tzwork@hotmail.com; Tel: 86-10-84931804
† These authors contributed equally to this work.

Table S1 Specific surface areas of MWNTs and HMWNTs prior to and after adsorbed HA

Samples	OD (nm)	BET (m^2 g^{-1})	BET after adsorption (m^2 g^{-1})	The rate of reduction of BET* (%)
MWNTs	4–6	495.52	342.41	30.90
	5–15	319.88	213.78	33.17
	20–30	161.65	129.49	19.89
HMWNTs	4–6	549.72	471.58	14.21
	5–15	250.40	220.42	11.97
	20–30	181.76	135.11	25.67

* The decreased rate of the specific surface area (BET) of CNTs after adsorption of HA.

Table S2 Parameters of Temkin and Dubinin-Radushkevich adsorption isotherms for HA adsorption on MWNTs and HMWNTs

Samples	Temkin			Dubinin-Radushkevich		
	a_t	b_t	R^2	q_m	$k \times 10^{-5}$	R^2
MWNT-1	0.252	82.42	0.958	61.99	1.032	0.932
MWNT-2	0.398	157.01	0.929	3.23	0.291	0.832
MWNT-3	0.178	157.61	0.933	29.25	1.921	0.803
HMWNT-1	0.290	218.67	0.928	21.67	0.566	0.670

HMWNT-2	0.253	206.98	0.975	22.56	0.824	0.840
HMWNTs-3	0.252	219.45	0.921	20.78	0.614	0.594

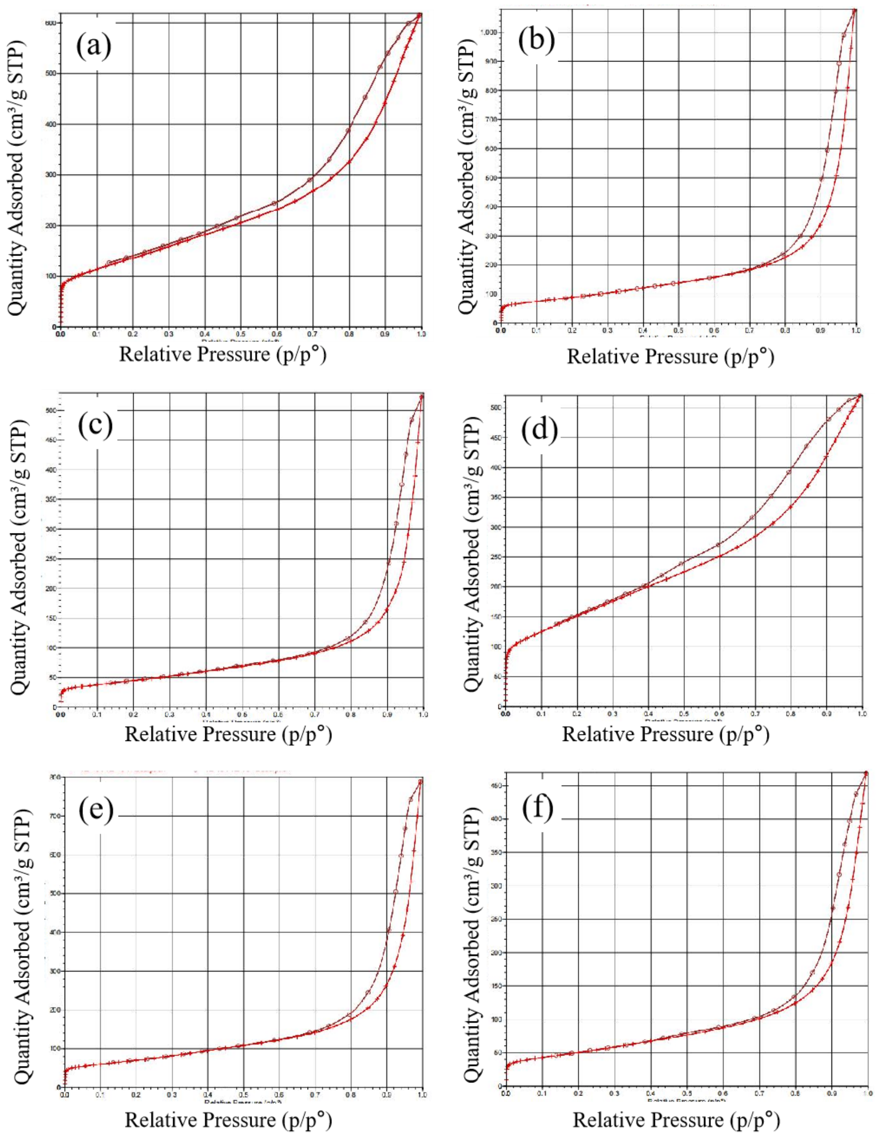

Figure S1. Nitrogen adsorption isotherms of MWNTs (a)(b)(c) and HMWNTs(d)(e)(f)

To better understand the adsorption behavior of HA on the surfaces of MWNT-1 and MWNT-2, we also used Temkin (Eq. (1)) and Dubinin-Radushkevich (Eq. (2), (3)) adsorption isotherms to analyze the adsorption of HA shown in Figure S2 and Figure S3.

$$q_e = \frac{RT}{b_t}\ln a_t + \frac{RT}{b_t}\ln C_e \tag{1}$$

$$\ln q_e = \ln q_m - k\varepsilon^2 \tag{2}$$

$$\varepsilon = RT\ln(1 + \frac{1}{C_e}) \tag{3}$$

where k is a constant related to the adsorption amount ($mg^2\ mg^{-2}$), a_t and b_t are constants of the equation. R is the ideal gas constant (8.314 J $(mol\ K)^{-1}$), T (K) is the thermodynamic temperature, and ε is the adsorption potential.

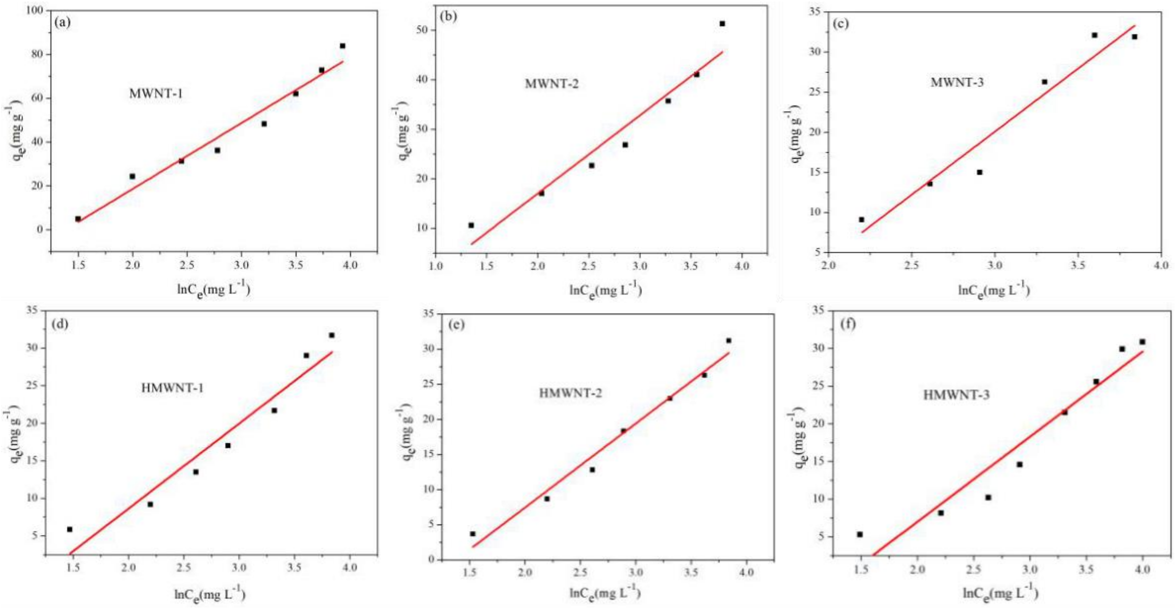

Figure S2. Temkin adsorption isotherm of HA adsorption on MWNTs(a)(b)(c) and HMWNTs(d)(e)(f)

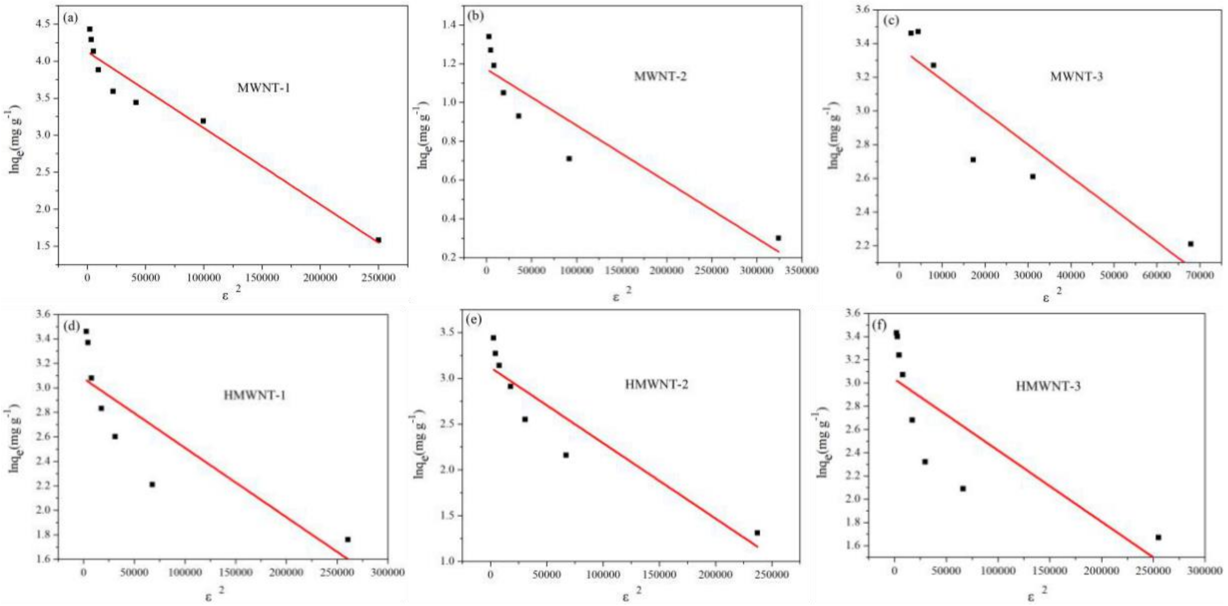

Figure S3. Dubinin-Radushkevich adsorption isotherm of HA adsorption on MWNTs (a)(b)(c) and HMWNTs(d)(e)(f)

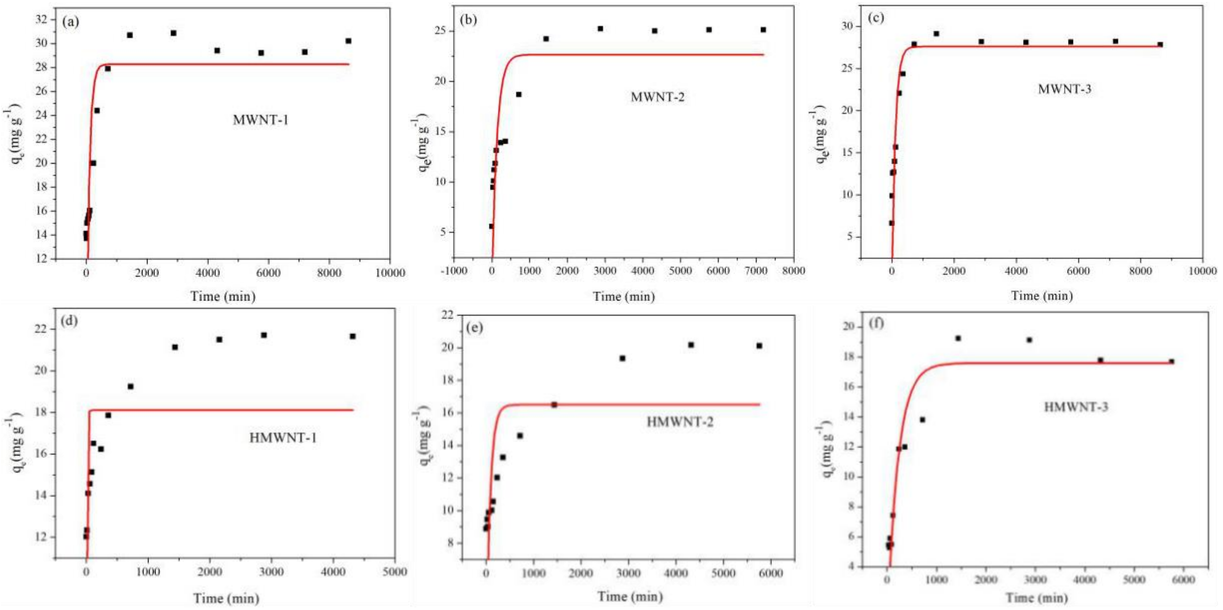

Figure S4. Pseudo-first-order kinetic curves of adsorption of HA on the surface of MWNTs (a)(b)(c) and HMWNTs(d)(e)(f)

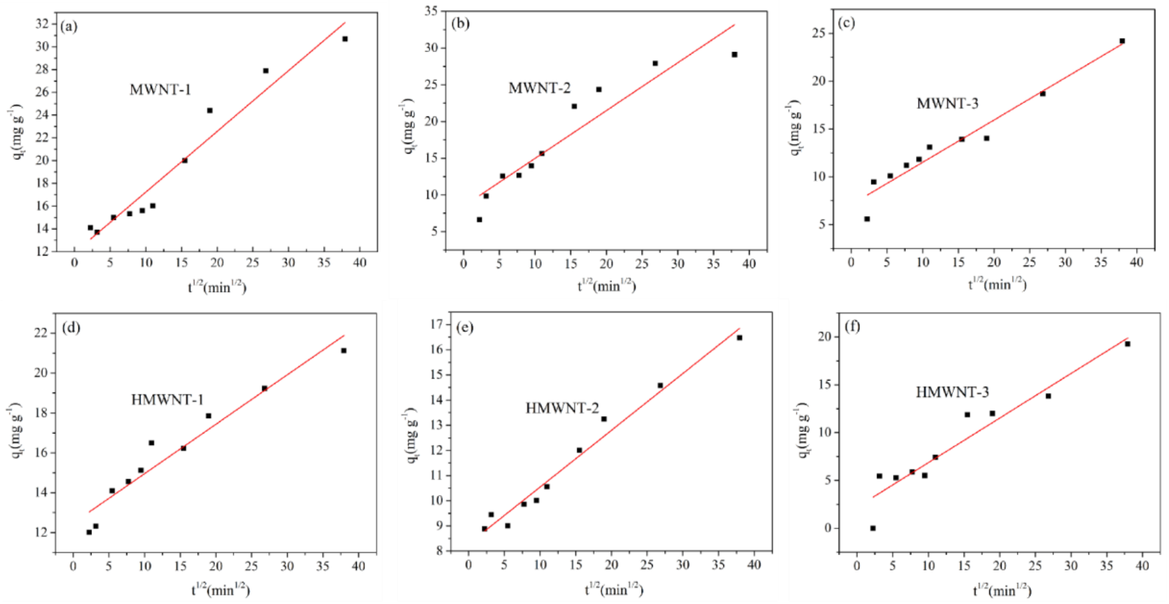

Figure S5. Plots of the intraparticle diffusion kinetic curves of adsorption of HA onto MWNTs (a)(b)(c) and HMWNTs(d)(e)(f)

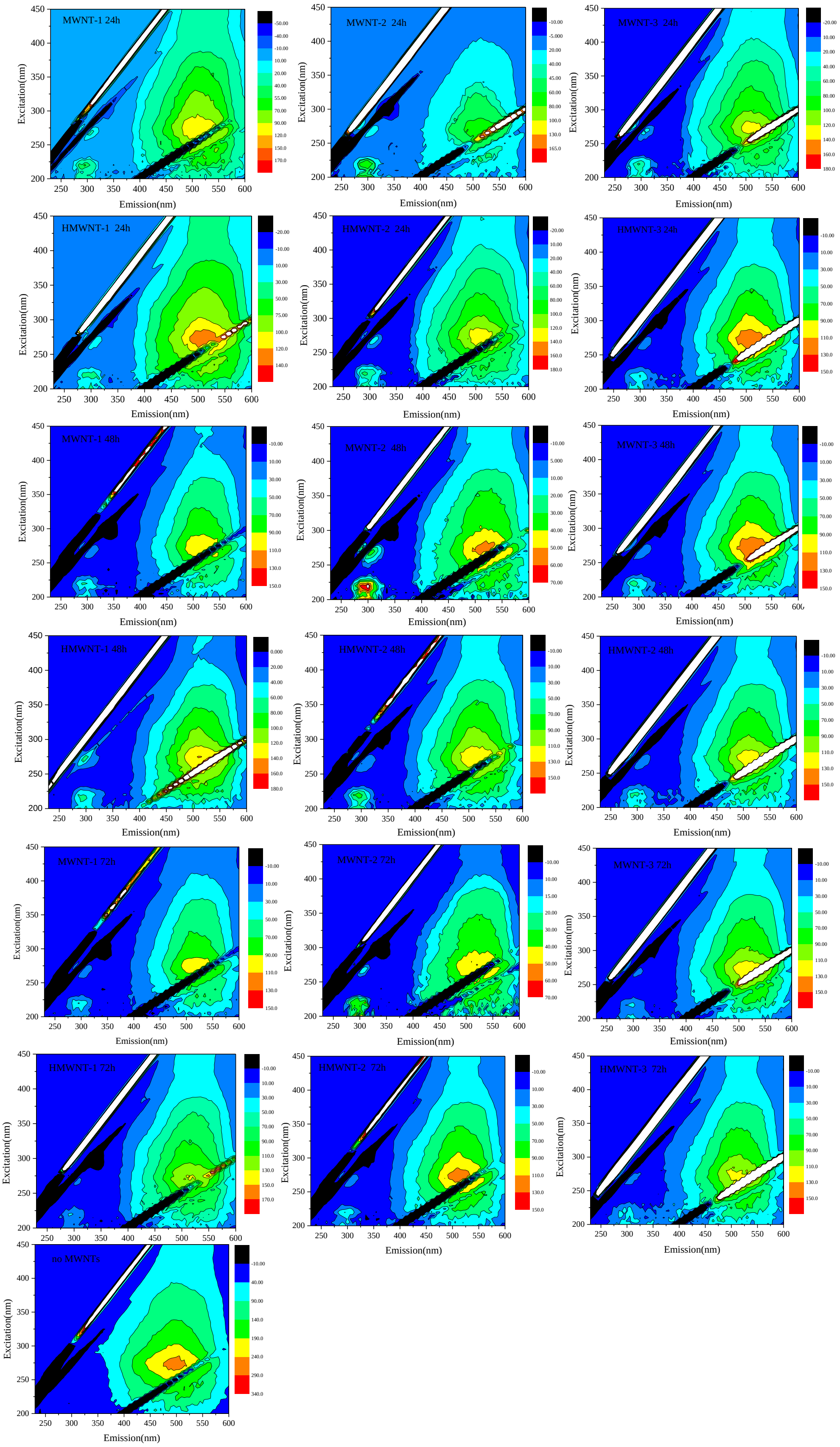

Figure S6. Fluorescence excitation-emission matrices (EEM) peaks for HA after adsorption of MWNTs and HMWNTs
